# Practical Notes and Formulæ

**Published:** 1887-04

**Authors:** 


### PRACTICAL NOTES AND FORMULÆ.

**Remedy for Whooping Cough.**—Dr. Haskell, (in *Medical World*) says: In a recent journal there is a call for a prophylatic for whooping cough. I do not know of one; but can mention a remedy which, after fifteen years of trial, I consider *almost a specific* for the disease in its early stage; and one also which, given before or immediately after the first symptoms of whooping cough, causes the disease to take a shorter and milder course than it would otherwise take.

I allude to sulphuret of potassium. There will be little or no improvement for four or five days. Then the improvement will be marked and the patient will usually be well in two weeks.

The article used must be fresh or it will be useless. It must have a strong odor of sulphuretted hydrogen (rotten eggs). The taste is pleasant and it is usually taken willingly by children:

℞. Potass. sulphuret..............................ʒ j,

Aquæ..............................℥ vss,

Syrupi..............................f℥ ijss,

Ess. cinnamomi..............................ʒ j.

M. Sig.—One to 4 teaspoonfuls three or four times a day. To a child of one year give a teaspoonful at a dose, and increase the dose one teaspoonful for each year up to four years. Then increase half a teaspoonful for each year above four.

I *have little doubt* of this remedy as a prophylactic, but have never tested it as such.

**For Acute Bronchitis.**—

℞. Ext. yerba santæ fl.,

Ext. grindeliæ rob. fl., } aa..............................f℥ j,

Syrup tolutan..............................f℥ vj.

M. Sig.—Dose, f ʒ j to f ʒ ij, every hour or two as needed for cough.—*Therapeutic Gazette.*

**Hypodermic Medication in Severe Uterine Hemorrhage.**—

℞. Ext. ergotæ aq..............................3 parts,
Glycerini..............................7 parts,
Aq. dest..............................7 parts.

M. Sig.—Twenty minims to be injected in the thigh or abdomen.—*Medical World.*

**Inflammation of the Hair Follicles within the Nares.**—Dr. Hardway, in *Journal Cutaneous and Veneral Diseases*, says of this annoying affection: As regards the internal treatment, and such treatment I have found nearly always to be necessary, it is well to correct any obvious errors of health, especially inquiring into matters of digestion, and then to institute a general tonic course. The following combination has appeared to me particularly serviceable:

℞. Ol. morrhuæ … fl. ℥ iv,  

Pancreatin saccharat … ʒ i,  

Pul. acaciæ … q. s,  

Glyceriti hypophospiti, Syr. calcii lactophosphatis, Aquæ, aa … fl. ℥ iv,  

Ol. gaultheriæ … gtt. xxx.

M. et ft. emulsion. Sig.—Tablespoonful three times a day after meals.

I generally precede this, however, with the sulphide of calcium for a few days, giving one-tenth grain every third hour.

**T. A. Edison's Anæsthetic.**—

℞. Chloral hydrat … 30 grams,  
Sp. vin … 110 grams,  
Chloriformi … 90 grams,  
Camphoræ … 60 grams,  
Ol. menth. pip … gtt. v,  
Ol. caryophylli … gtt. v,  
Etheris … 50 grams,  
Acid salicylici … 5 grams,  
Amyl. nitrit … 3 grams,  
Morphin. sulph … 2 grams.  
M.

The celebrated inventor thought so highly of this remedy, that he had it patented in England. He used it as a liniment in neuralgia and other painful affections.—*Ibid.*

**For Cancer of the Stomach.**—Germain See gives the following for the relief of the pain after taking food, in patients with cancer of the stomach:

℞. Tinct. conii, Tinct. hyoscyami, Ol. anisi, aa … ʒ ij,  

Tinc. gentianæ … ʒ j.

S.—Ten to 30 drops after meals.—*Medical Age.*

**Camphorated Dover's.**—The following is Beech's diaphoretic powder:

℞. Powdered opium … grs. x,  
Powdered camphor … grs. xxxx,  
Powdered ipecac … grs. xx,  
Powdered bitartrate of potassium … grs. clx.  
M.

**Terebene.**—A remedy for cough, in doses of five drops. It is also used successfully as antiseptic.

**Pills for Phthisis.**—Potain recommends the following:

℞. Creasote . . . . . . . . . . . . . . . . . . . . . . . . . . . . . . *m* ½,

Iodoform . . . . . . . . . . . . . . . . . . . . . . . . . . . . . gr. 1.10,

Ext. of opium . . . . . . . . . . . . . . . . . . . . . . . . . . gr. ¼,

Balsam of tolu, } aa . . . . . . . . . . . . . . . . . . . . . . *m* ⅓.

Turpentine, }

M.

Of these from 3 to 10 may be taken daily.

**Treatment of Syphilis.**—In answer to Dr. C. A. Holt, Troy, N. Y., I would say I have within the last thirty years had a great many cases of syphilis. For the last fifteen years I have not failed to cure every case, with the following prescription. One case had been under treatment for six years with calomel, iodide of potash and sarsaparilla, etc., etc.

℞. Fluid ext. of smilax sylvatica, }
Fluid ext. of lappa minor, } aa . . . . . . . . . . . . . . ℥ ij,
Fl. ext. phytolacca decandra, }
Tinct. xanthoxylum . . . . . . . . . . . . . . . . . . . . . . . . ℥ j,
M. If there are gummata, add iodide pot . . . . . . . . . . . . ℥ j,

Sig.—One teaspoonful to ʒ j of cold water before meals, and gradually increase to a tablespoonful.—*Dr. J. B. Sullivan, in Medical World.*

**For Hysteria.**—

℞. Tr. asafœtidæ . . . . . . . . . . . . . . . . . . . . . . . . . . . f ℥ ij,

Tr. lobeliæ, } aa . . . . . . . . . . . . . . . . . . . . . . . . . f ℥ j,

Tr. opii camph., }

Aetheris . . . . . . . . . . . . . . . . . . . . . . . . . . . . . . . . f ℥ j,

M. Sig.—Dose, one teaspoonful every hour until four teaspoonfuls are taken, when in nine out of ten cases the paroxysm is arrested.—*I. R. S., in Ibid.*

**For Chronic Bronchitis.**—

℞. Potas. iodid . . . . . . . . . . . . . . . . . . . . . . . . . . . . . ʒ ij,

Ammon. iodid . . . . . . . . . . . . . . . . . . . . . . . . . . . . ʒ j,

Potas. carb . . . . . . . . . . . . . . . . . . . . . . . . . . . . . . ʒ jss,

Syr. rhei arom., ad . . . . . . . . . . . . . . . . . . . . . . . . ℥ iij.

M. Sig.—Dose, f ʒ j every four hours, in hot water.

**Dr. Rorick's Formula for Injecting Hemorrhoids.**—

℞. Acid. carbolic, } aa . . . . . . . . . . . . . . . . . . . . . . . f ʒ ij,

Glycerini, }

Aquæ . . . . . . . . . . . . . . . . . . . . . . . . . . . . . . . . . . f ʒ jss,

Ext. ergotæ fl . . . . . . . . . . . . . . . . . . . . . . . . . . . . f ʒ j.

M. Sig.—Three to 8 drops to be injected, according to size of tumor.—*Chicago Medical Times.*

